# National Hospitalization Trends and the Role of Preventable Hospitalizations among Centenarians in the United States (2000–2009)

**DOI:** 10.3390/ijerph19020795

**Published:** 2022-01-12

**Authors:** Sylvia E. Twersky, Adam Davey

**Affiliations:** 1 Department of Public Health, The College of New Jersey, Ewing, NJ 08628, USA; 2 Department of Behavioral Health and Nutrition, University of Delaware, Newark, DE 19716, USA; davey@udel.edu

**Keywords:** centenarians, preventable hospitalizations, health care costs, racial disparities

## Abstract

Increases in life expectancy mean that an unprecedented number of individuals are reaching centenarian status, often with complex health concerns. We analyzed nationally representative hospital admissions data (200–2009) from the National Inpatient Study (NIS) for 52,618 centenarians (aged 100–115 years, mean age 101.4). We predicted length of stay (LOS) via negative binomial models and total inflation adjusted costs via fixed effects regression analysis informed by descriptive data. We also identified hospitalizations due to ambulatory care-sensitive conditions defined by AHRQ Prevention Quality Indicators. Mean LOS decreased from 6.1 to 5.1 days, while over the same time period the mean total adjusted charges rose from USD 13,373 to USD 25,026 in 2009 dollars. Black, Hispanic, Asian, or other race centenarians had higher cost stays compared to White, but only Black and Hispanic centenarians had significantly greater mean length of stay. Comorbidities predicted greater length of stay and higher costs. Centenarians admitted on weekends had higher costs but shorter length of stay. In total, 29.4% of total costs were due to potentially preventable hospitalizations for total charges (2000–2009) of USD 341.8M in 2009 dollars. Centenarian hospitalizations cost significantly more than hospitalization for any other group of elderly in the U.S.

## 1. Introduction

Centenarians are one of the fastest growing demographic age groups in the United States. In 2010, there were 53,364 people aged 100 and older in the U.S. and this number jumped to 72,197 in 2014 [1,2]. Individuals are living longer with chronic diseases, meaning that an unprecedented number of individuals are reaching centenarian status with complex health concerns. Little research has addressed the hospitalization experiences of centenarians. It is particularly important to elucidate the role of preventable hospitalizations since these could indicate a lack of adequate primary care for this particularly vulnerable population and are costly to both the individual and the healthcare system. 

Heart disease, Alzheimer’s disease, stroke, cancer, and influenza and pneumonia were the leading causes of death for centenarians in 2014, with heart disease having an outsize effect accounting for 34.6% of centenarian deaths [2]. A study of participants in the Health and Retirement Survey participants, who reached the age of 100 in 2010, showed that only 23% reached that age with no chronic disease [3]. The vast majority of centenarians are living with chronic conditions as they become the oldest of the old [4,5], and these conditions are likely to multiply over time. A study looking at people aged 70–115 years being treated in the VA for cancer found that 70% had hypertension, over 50% had hyperlipidemia, 40% had heart disease, 25% had diabetes, and 21% had osteoarthritis, with the oldest old having the greatest prevalence of comorbidities [6]. An increase in the number of hospitalizations of centenarians in the U.S. has been observed between 2004 and 2008, and 96.9% of those admitted had mild or moderate comorbidities [7]. Evidence indicates that centenarians are likely to be living with multiple chronic conditions, potentially complicated by frailty and changes to mental status, that if not adequately addressed in an ambulatory care setting could lead to preventable hospitalizations. 

Preventable hospitalizations are defined as potentially avoidable admissions to the hospital for an acute condition or worsening chronic condition that can be managed in an ambulatory care setting and can be used as a proxy for access and quality of ambulatory care systems [8]. These preventable hospitalizations are defined according to the Agency for Healthcare Research and Quality (AHRQ) and include diabetes, perforated appendix, chronic obstructive pulmonary disease (COPD) and asthma, hypertension, heart failure, dehydration, bacterial pneumonia, urinary tract infections, angina without procedure, and lower extremity amputations among patients with diabetes. Interestingly, a study using a subsample of Medicare claims data found that per capita spending for Medicare recipients in 2011 peaked at the age of 96 and then declined for beneficiaries 97 and above. This was also reflected in inpatient hospitalization costs to Medicare where individuals 95 and older cost less than the 75–94 age groups [9]. This may indicate the costs for preventable hospitalizations may actually be lower among centenarians compared to a 65–99 aged group. However, any costs associated with preventable hospitalizations among the centenarian group reflects an unused cost saving for the healthcare system, as this group is insured primarily through Medicare-federal old age insurance- and Medicaid-state and federally funded insurance for low-income populations. 

There is a disparity in the racial and ethnic makeup of the centenarian population in the U.S., with 82.5% identifying as White in the 2010 census compared to 72.4% in the total U.S. population. Among centenarians in 2010, 5.8% identity as Hispanic or Latino compared to 16.3% in the total population. In total, 12.2% were Black or African American, 2.5% Asian, and 2.3% some other race or more than one race in 2010 [1]. Racial and ethnic minorities are less likely to survive to become centenarians. A 2001–2009 study of the National Inpatient Study (NIS) data showed that while potentially preventable hospitalization rates declined over that time period overall, Black or African American adults had over twice the rate of potentially preventable hospitalizations compared to the white non-Hispanic population and the rates of preventable hospitalization were consistently higher among the Hispanic population as well [8]. There is some question as to whether survivorship into the oldest age group may attenuate differences in health outcomes by race and ethnicity [10], but data are scanty. Specifically, among the 65 plus population in 2014 using Medicare claims data 15% of White, 18% of Black, and 17% of Hispanic patient’s hospitalizations were potentially preventable [11]. This data indicates that disparities in health outcomes persist into the oldest old age groups.

## 2. Methods

For this analysis we used nationally representative hospital admissions data (2000–2009) from the NIS. There is some evidence that Medicaid expansion reduced preventable hospitalizations [12], so we chose a time period for analysis before the implementation of the Affordable Care Act in 2010. In addition, the latest census data available describing the centenarian population in the U.S. is the 2010 census. Furthermore, it is key to analyze historical data in order to understand trends over time. The NIS was developed for the Healthcare Cost and Utilization Project (HCUP) and is the largest publicly available all-payer inpatient healthcare database with discharge-level healthcare data. It was designed to produce regional and national estimates of inpatient utilization, access, charges, quality, and outcomes. We included data for all centenarians who had an in-patient hospitalization in that time period, regardless of admissions source.

### 2.1. Measures

Our primary outcome measures were as follows: (1) total charges in USD and (2) length of stay in days. Charges were adjusted for general medical price change using the Personal Consumption Expenditure Health Price Index [13] with 2009 as reference year and were natural log transformed prior to analysis. Charges are for the hospital cost of producing the services and do not include physician charges associated with a hospital stay. Length of stay was coded as days between admission and discharge.

Age was measured in years. Sex was coded as female = 1, male = 0. Race codes included identification as 1 = White (reference group), 2 = African American, 3 = Hispanic, 4 = Asian or Pacific Islander, and 5 = Other. Number of recorded diagnoses was used as an indicator of multimorbidities. Whether an admission occurred over the weekend (1 = yes, 0 = no) was coded as this may impact the length of stay. The NIS coded whether the patient died during their hospital stay and this was included in the models as a covariate (1 = dead, 0 = alive) in order to determine whether there was a cost differential for individuals who died in the hospital during that stay. Whether the patient was admitted from the emergency department (1 = yes, 0 = no) was coded to understand if it was an emergent hospitalization. We coded whether an individual was hospitalized due to potentially preventable conditions defined by AHRQ guidelines as a primary diagnosis (1 = yes, 0 = no), as defined in the introduction above. Finally, we coded the admission year (2000–2009).

### 2.2. Statistical Analysis

Sample descriptive statistics were estimated separately for men and women and are presented in Table 1. The most common primary diagnoses, average number of comorbidities, payer types, and demographics for the centenarian patients are presented in the descriptive table.

Total costs were inflation-adjusted to 2009 USD-equivalents prior to analysis. They were also first natural log transformed (adding 1 so that the zero point would be equivalent in transformed and untransformed analyses). Models for ln(Total Charges + 1) evaluated the predictor of interest (potentially preventable hospitalizations) and adjusted for the following potential confounders: age, sex, dummy coded race, length of stay, number of diagnoses as an indicator of multimorbidities, weekend admission, admission from emergency department, whether the patient died during the hospital stay, and dummy coded year (to capture non-linear secular trends).

Models for length of stay evaluated the predictor of interest (potentially preventable hospitalizations or not) and adjusted for the following potential confounders: age, sex, dummy coded race, length of stay, number of diagnoses as an indicator of multimorbidities, weekend admission, admission from emergency department, whether the patient died during the hospital stay, and dummy coded year (to capture non-linear secular trends).

Based on the results of Hausman specification tests, both outcomes were estimated via fixed effects models to address unobserved heterogeneity. Natural log transformed total costs were modeled via fixed effects regression and length of stay was estimated using a fixed effects negative binomial model to allow for overdispersion. Both models adjusted for clustering at the level of the state.

## 3. Results

### 3.1. Sample Descriptive Characteristics

Table 1 presents descriptive statistics for our sample (n = 52,618; aged 100–115 years; mean age 101.4 years) separately by men and women on all study variables. As reflected in the general population of centenarians, the number of women in the sample greatly outweigh the number of men. Among the centenarians that were hospitalized between 2000 and 2009, 75.8% identified as White, 13.6% as Black, 5.8% as Hispanic, 2.3% as Asian and Pacific Islander, and 2.4% identified as other race. The primary payer for hospital stays was Medicare with only 4% of the men and 0.7% of the women self-paying. Government sponsored insurance (Medicare and Medicaid) was the primary payer in 90.7% of the centenarian hospitalizations. On average, the centenarians had eight diagnoses upon admission to the hospital, demonstrating the high level of multimorbidity typical for this age group [14].

Table 2 presents the 10 most common overall diagnoses by sex. Nine of the top 10 diagnoses were common to men and women. Pneumonia was the most frequent diagnosis, followed by congestive heart failure and urinary tract infections. Gastrointestinal hemorrhage was a leading diagnosis among female but not male centenarians, while intercranial hemorrhage was in the top 10 for males only. 

### 3.2. Total Charges

Table 3 shows the total and mean adjusted charges per centenarian hospitalizations in 2009 USD by year and by potentially preventable hospitalizations. While the mean centenarian hospitalization costs was USD 22,308 over the years 2000–2009, the range was a low of USD 18,833 in 2000 to a high of USD 25,027 in 2009. The total national charges for centenarian hospitalizations in this time period was USD 116 billion, of which USD 34.2 billion was due to a potentially preventable hospitalization as a primary diagnosis at admittance to the hospital. Mean adjusted total charge per hospitalization over this time period was highest for centenarians identifying as other race and ethnicity while the lowest mean charges were for Asian or Pacific Islander centenarians. On average, the total adjusted charges per hospitalization were lower for female centenarians than for males.

Results from the fixed-effects regression model for ln (total charges + 1) are shown in Table 4. Total charges were higher on average for older centenarians (*p* ≤ 0.05) and lower for women than men (*p* ≤ 0.0001). Compared to Whites, total charges were significantly higher for Black, Hispanics, Asian and Pacific Islanders, and other race centenarians. Length of stay (*p* ≤ 0.0001) and number of diagnoses (*p* ≤ 0.0001), weekend (*p* ≤ 0.05) or emergency department admission (*p* ≤ 0.0001) was associated with significantly higher total charges. In contrast, total costs were lower for individuals who died during their hospital stay and those centenarians admitted with an ambulatory care sensitive diagnosis. Costs (in adjusted dollars) were significantly higher than in 2000 for every year except 2001.

Table 5 shows the adjusted total charges per hospital stay for each of the top 10 primary diagnoses for each year. Even after adjustment to 2009 dollars, there is still a distinct upward trend in total charges for each diagnosis, consistent with the results of the multivariable regression model. Diagnoses with an asterisk in Table 4 indicate potentially preventable hospitalizations. 

### 3.3. Length of Stay

Results from the fixed-effects negative binomial regression model for length of stay are shown in Table 6. Length of stay was slightly but significantly shorter among older centenarians. Compared with White centenarians, Black (*p* ≤ 0.0001) and Hispanic (*p* ≤ 0.05) centenarians had longer average stays. Individuals with multimorbidities had significantly longer stays, whereas individuals admitted on weekends or who died during hospitalization had shorter stays. Lengths of stay were significantly shorter than in 2000 for every year except 2001 (*p* ≤ 0.0001).

Table 7 shows mean length of stay for each of the top 10 primary diagnoses for each year. There is still a distinct trend toward shorter stays for each diagnosis, consistent with the results of the multivariable negative binomial model. The rows with asterisks indicate ambulatory care sensitive diagnoses and associated length of hospital stay for centenarians. 

## 4. Discussion

We set out to characterize hospitalization trends among centenarians in the decade prior to the implementation of the Affordable Care Act. Increases of life expectancy of 2.2 years per decade of life indicate that the proportion of individuals reaching centenarian status is likely to increase dramatically in the coming decades [15]. At the same time, there are mounting concerns about the burden of disease and associated costs that a large proportion of these individuals are likely to encounter in their second century of life [16].

Our analyses suggest that health disparities by race persist even among exceptional survivors. Compared with Whites, mean adjusted total charges were USD 4057 higher for Blacks, USD 12,962 more expensive for Hispanics, USD 15,189 more expensive for Asian or Pacific Islanders, and USD 8498 more expensive for individuals reporting other race. While Black, Hispanic, Asian or Pacific Islanders, and other race centenarians had higher cost stays compared to White, only Black and Hispanic centenarians had significantly longer mean hospital stays. This indicates that other race centenarians had other drivers besides length of stay for the higher cost per hospitalizations. 

Comorbidities predicted both greater length of stay and higher costs. Centenarians admitted on weekends had higher costs but shorter lengths of stay per hospitalization. The length of the hospital stay for centenarians decreased on average from 2002 through 2009, but adjusted total charges increased over the same time period. Therefore, while centenarians were staying for a reduced length of time in the hospital per admission, the cost if that admission in adjusted dollars is actually increasing. This has real implications for Medicare, the primary payer for centenarian hospitalizations. 

We also found evidence of significant sex differences in hospitalization charges, such that the average adjusted total charge of stay was USD 5070 higher for men than women, despite comparable lengths of stay. This may be explained in part by women being functionally younger at every age in the centenarian population [10]. This finding is consistent with general population hospitalization costs, where on average the hospital stay for women costs less than for men [17].

Centenarian hospitalizations cost significantly more than hospitalizations for other groups of older adults in the U.S. Centenarians (USD 25,027) had 2.7 times the average hospital costs of the 85 plus population (USD 9400) and 2.1 times the costs of the 65–84 (USD 11,900) population in 2009 [17]. One study in the U.K. has suggested that total healthcare costs among individuals aged 80 and above may be highest in the last year of life [18]. In the current analyses, approximately 10.8% of centenarians died in the hospital, so this is unlikely to drive the current findings alone. However, our results suggest that there are considerable sex differences in costs by discharge status (alive/dead). Specifically, total charges (2009 USD) for male centenarians were USD 25,000 when alive at discharge and USD 36,172 when deceased at discharge. Total charges (2009 USD) for female centenarians were USD 20,916 when alive at discharge and USD 24,746 when deceased at discharge. Differences in age-graded hospitalization charges among the oldest old is a topic worthy of further consideration in light of sex differences that are even greater among the centenarian population than individuals 85+.

Hospitalizations due to ambulatory care-sensitive conditions as primary diagnoses are a significant contributor to admissions and cost in the centenarian population. Approximately 29% of total hospitalization charges and 34% of all admissions were associated with potentially preventable hospitalizations for a total charge (2000–2009) of USD 34.2 billion in 2009 dollars.

### 4.1. Limitations

Application of this study globally may not be possible as there have been shown to be differences between the health status of U.S. centenarians and centenarians in other countries [19,20]. While this data is potentially informative, cross-national comparisons are difficult due to differences in health status, health systems, and policies. 

The period of data analysis was selected to align with both with a period immediately prior to implementation of important relevant national policy as well as the most current data available on centenarians from the Census. Expanding the analyses to consider the period immediately following implementation of the ACA, which includes many pilot projects on medical care delivery such as Accountable Care Organizations, will thus be an important next step.

As centenarians remain so rare even among the older adult population, the granularity of data that we would wish for to understand the needs of this exceptional population, particularly at the community level, is unavailable. For this reason, we currently have few tools available to answer questions such as what proportion of centenarians is hospitalized in any given year, the proportion of centenarians with multiple hospitalizations, and what characteristics distinguish centenarians with potentially preventable diagnoses who are hospitalized from those who do not. To address these questions, data on both hospitalized and non-hospitalized centenarians would be needed.

This data, while showing a significant impact of preventable hospitalizations on cost among the centenarian population, does not allow us to ascertain the specific drivers behind these potentially preventable hospitalizations such as lack of access to care and/or the quality of the care provided.

### 4.2. Future Directions

Future analyses can use these historical data trends in order to understand the impact of changing policies and demographics on preventable hospitalizations and associated costs.

## 5. Conclusions

More than one third of centenarian hospitalizations are for potentially preventable ambulatory care diagnoses. Since government sponsored insurance (Medicare and Medicaid) is the primary payer in this population in over 90% of hospitalizations and the of people who survive into their 10th decade in the U.S. are increasing, this is an area to leverage potential savings through greater access to quality primary care for centenarians. It is also important to recognize that racial disparities in health outcomes persist even among exceptional survivors. 

## Figures and Tables

**Table 1:** Centenarian characteristics of 2000–2009 hospitalizations.

	Men (*n* = 10,472)		Women (*n* = 42,146)	
	Mean	SD	Mean	SD
Age	101.5	2.1	101.4	1.7
Race and Ethnicity				
White	72.3%	0.448	76.7%	0.423
Black	12.2%	0.327	14.0%	0.347
Hispanic	8.4%	0.277	5.1%	0.221
Asian or Pacific Islander	3.7%	0.190	2.0%	0.141
Other	3.4%	0.180	2.2%	0.147
Primary Payer				
Medicare	83.8%	0.386	93.2%	0.014
Medicaid	2.9%	0.167	1.4%	0.119
Private Insurance	7.2%	0.259	4.0%	0.196
Self-Pay	4.0%	0.197	7.0%	0.082
Primary Diagnosis				
Angina	0.1%	0.024	0.1%	0.030
Asthma	0.4%	0.062	0.4%	0.061
Cellulitis	0.1%	0.029	0%	0.018
Chronic obstructive pulmonary disease	1.3%	0.112	1.1%	0.104
Congestive heart failure	7.2%	0.259	9.1%	0.288
Dehydration	3.8%	0.191	4.4%	0.205
Diabetes	0.5%	0.070	0.4%	0.063
Gastroenteritis	0.3%	0.051	0.4%	0.061
Epilepsy	0.4%	0.065	0.3%	0.056
Hypertension	0.7%	0.083	0.9%	0.096
Hypoglycemia	0.4%	0.063	0.4%	0.063
Urinary tract infection	4.0%	0.195	6.2%	0.241
Pneumonia	12.7%	0.334	11.2%	0.316
Enteric Infection	1.1%	0.103	0.8%	0.088
Avg. Number of Diagnoses	8.3	3.9	8.2	3.5

**Table 2:** Leading diagnoses among hospitalized centenarians in 2000–2009.

Diagnosis	Men	Women
Pneumonia	1335	4729
Congestive heart failure	759	3838
Urinary tract infections	416	2616
Fracture of neck of femur	407	2475
Septicemia	433	1883
Fluid and electrolyte disorders	399	1845
Aspiration pneumonitis	527	1335
Acute cerebrovascular disease	302	1484
Acute myocardial infarction	321	1265
Gastrointestinal hemorrhage	-	1163
Intracranial injury	248	-

**Table 3:** Inflation adjusted total charges for centenarian hospitalizations from 2000 to 2009.

All Charges in 2009 USD	Mean (USD)	SE	Total in Billion USD
2000–2009	22,308	143	116.0
2000	18,833	504	8.8
2001	18,836	372	9.1
2002	20,944	458	10.4
2003	21,296	464	11.0
2004	21,749	424	10.9
2005	23,736	505	12.4
2006	22,285	340	12.5
2007	24,318	459	12.8
2008	24,869	386	14.5
2009	25,026	567	1.37
ACS Primary Diagnosis	18,857	169	34.2
Non ACS Primary Diagnosis	24,153	199	81.9
White	22,428	191	66.2
Black	26,486	572	14.1
Hispanic	35,390	958	7.9
Asian or Pacific Islander	37,618	1725	3.4
Other	30,927	1447	2.6
Male	26,373	436	27.2
Female	21,303	142	88.9

**Table 4:** Factors that predict inflation adjusted total charges per hospital visit among centenarians.

ln (Total Charges + 1)	b	SE(b)	t	*p*-Value
Age	0.005	0.002	2.4	0.016
Female	−0.071	0.009	−8.0	<0.0001
Race				
Black	0.129	0.011	11.8	<0.0001
Hispanic	0.147	0.016	9.3	<0.0001
Asian or Pacific Islander	0.127	0.026	4.9	<0.0001
Other	0.115	0.023	4.9	<0.0001
Length of Stay	0.036	0.000	84.3	<0.0001
Num. of Diagnoses	0.065	0.001	63.0	<0.0001
Weekend Admission	0.020	0.008	2.4	0.017
Died	−0.133	0.011	−11.6	<0.0001
Admitted from Emergency Dept.	0.234	0.010	24.5	<0.0001
ACS Primary Diagnosis	−0.106	0.007	−14.2	<0.0001
Year				
2001	0.027	0.017	1.6	0.111
2002	0.073	0.017	4.3	<0.0001
2003	0.143	0.017	8.6	<0.0001
2004	0.168	0.017	10.0	<0.0001
2005	0.169	0.017	10.1	<0.0001
2006	0.139	0.016	8.5	<0.0001
2007	0.168	0.017	10.0	<0.0001
2008	0.137	0.016	8.4	<0.0001
2009	0.098	0.017	5.8	<0.0001
Intercept	8.209	0.202	40.7	<0.0001

**Table 5:** Adjusted total charges for centenarians per hospitalization by primary diagnoses.

Adj. Total Charges	2000	2001	2002	2003	2004	2005	2006	2007	2008	2009
Pneumonia *	18,854	19,162	20,232	20,466	20,880	21,811	21,847	24,182	23,096	23,014
Congestive heart failure *	15,528	16,095	15,907	17,866	18,657	20,034	20,302	20,939	19,714	21,726
Urinary tract infections *	14,060	13,654	16,129	14,879	19,006	17,462	17,466	18,748	18,336	16,056
Fracture of neck of femur *	25,452	27,691	30,386	33,871	35,365	36,151	35,509	36,350	42,915	40,539
Septicemia	25,254	27,292	25,056	27,623	23,604	37,999	29,825	41,361	39,656	33,683
Fluid and electrolyte disorders *	12,740	13,035	14,630	14,420	16,340	15,420	15,183	16,531	18,494	16,389
Aspiration pneumonitis *	29,204	36,667	35,830	32,325	30,786	35,182	24,619	33,543	33,413	36,838
Acute cerebrovascular disease	19,844	19,987	16,960	17,528	22,657	21,775	21,621	21,992	23,677	23,831
Acute myocardial infarction	24,527	20,349	26,548	24,270	21,115	24,267	23,821	23,971	28,317	24,453
Gastrointestinal hemorrhage	14,864	14,359	19,370	19,147	14,936	20,844	17,720	22,253	18,389	18,648

* potentially preventable hospitalization.

**Table 6:** Factors that predict length of hospital stay among centenarians.

Length of Stay (Days)	b	SE(b)	z	*p*-Value
Age	−0.005	0.002	−2.6	0.008
Female	0.008	0.009	0.9	0.359
Race				
Black	0.099	0.011	9.4	<0.0001
Hispanic	0.051	0.016	3.3	0.001
Asian or Pacific Islander	−0.026	0.025	−1.0	0.304
Other	0.023	0.024	1.0	0.324
Num. of Diagnoses	0.060	0.001	63.1	<0.0001
Weekend Admission	−0.044	0.008	−5.3	<0.0001
Died	−0.216	0.012	−17.5	<0.0001
Admitted from ED	0.011	0.009	1.2	0.231
ACS Primary Diagnosis	−0.007	0.007	−1.0	0.328
Year				
2001	−0.001	0.017	−0.1	0.936
2002	−0.068	0.017	−4.1	<0.0001
2003	−0.070	0.016	−4.2	<0.0001
2004	−0.089	0.017	−5.4	<0.0001
2005	−0.121	0.017	−7.3	<0.0001
2006	−0.202	0.016	−12.4	<0.0001
2007	−0.230	0.017	−13.7	<0.0001
2008	−0.298	0.016	−18.3	<0.0001
2009	−0.349	0.017	−20.7	<0.0001
Intercept	1.086	0.205	5.3	<0.0001

**Table 7:** Average length of hospital stay for centenarians by primary diagnoses.

LOS	2000	2001	2002	2003	2004	2005	2006	2007	2008	2009
Pneumonia *	6.5	6.0	5.9	6.3	6.0	6.2	5.8	5.7	5.5	5.4
Congestive heart failure *	5.6	5.3	4.8	5.2	5.4	4.8	5.1	5.0	4.7	5.3
Urinary tract infections *	5.7	5.1	5.1	4.9	5.8	4.5	4.8	5.3	4.5	4.1
Fracture of neck of femur *	6.3	6.3	6.9	6.2	6.9	6.1	6.0	5.8	6.2	5.9
Septicemia	7.2	7.5	6.7	8.0	6.2	7.5	6.1	7.2	6.5	5.7
Fluid and electrolyte disorders *	5.4	5.0	4.9	4.8	4.9	4.5	4.3	4.4	4.5	4.6
Aspiration pneumonitis *	8.2	8.6	7.7	7.8	7.5	7.3	6.7	6.8	6.8	7.0
Acute cerebrovascular disease	9.0	5.9	5.5	5.2	5.8	5.5	4.6	4.7	5.2	4.8
Acute myocardial infarction	6.3	5.4	5.6	5.5	6.0	5.2	5.2	4.8	6.3	4.7
Gastrointestinal hemorrhage	5.1	6.0	5.1	4.7	4.0	4.6	4.1	4.5	3.9	4.3

* potentially preventable hospitalization.

## Data Availability

The Healthcare Cost and Utilization Project (HCUP) databases are available at http://www.ahrq.gov/research/data/hcup/index.html (accessed on 1 July 2011).
